# Resveratrol Modulates the Gut Microbiota and Inflammation to Protect Against Diabetic Nephropathy in Mice

**DOI:** 10.3389/fphar.2020.01249

**Published:** 2020-08-19

**Authors:** Ting-Ting Cai, Xiao-Long Ye, Ru-Run Li, Hui Chen, Ya-Yun Wang, Hui-Juan Yong, Ming-Lin Pan, Wei Lu, Ying Tang, Heng Miao, Antoine M. Snijders, Jian-Hua Mao, Xing-Yin Liu, Yi-Bing Lu, Da-Fa Ding

**Affiliations:** ^1^Department of Endocrinology, The Second Affiliated Hospital of Nanjing Medical University, Nanjing, China; ^2^Biological Systems and Engineering Division, Lawrence Berkeley National Laboratory, Berkeley, CA, United States; ^3^Department of Pathogen Biology-Microbiology Division, Key Laboratory of Pathogen of Jiangsu Province, Nanjing Medical University, Nanjing, China

**Keywords:** diabetic nephropathy, resveratrol, gut microbiome, inflammation, gut–kidney axis

## Abstract

Oral administration of resveratrol is able to ameliorate the progression of diabetic nephropathy (DN); however, its mechanisms of action remain unclear. Recent evidence suggested that the gut microbiota is involved in the metabolism therapeutics. In the current study, we sought to determine whether the anti-DN effects of resveratrol are mediated through modulation of the gut microbiota using the genetic db/db mouse model of DN. We demonstrate that resveratrol treatment of db/db mice relieves a series of clinical indicators of DN. We then show that resveratrol improves intestinal barrier function and ameliorates intestinal permeability and inflammation. The composition of the gut microbiome was significantly altered in db/db mice compared to control db/m mice. Dysbiosis in db/db mice characterized by low abundance levels of *Bacteroides*, *Alistipes*, *Rikenella*, *Odoribacter*, *Parabacteroides*, and *Alloprevotella* genera were reversed by resveratrol treatment, suggesting a potential role for the microbiome in DN progression. Furthermore, fecal microbiota transplantation, derived from healthy resveratrol-treated db/m mice, was sufficient to antagonize the renal dysfunction, rebalance the gut microbiome and improve intestinal permeability and inflammation in recipient db/db mice. These results indicate that resveratrol-mediated changes in the gut microbiome may play an important role in the mechanism of action of resveratrol, which provides supporting evidence for the gut–kidney axis in DN.

## Introduction

Diabetic nephropathy (DN) is the leading cause of end-stage renal disease, and therapeutic options for preventing its progression are limited. Several molecular mechanisms are involved in the pathophysiology of DN, including oxidative stress, the renin‐angiotensin system, transforming growth factor‐β (TGF‐β) activation, and the inflammatory response (Arora and Singh, 2013). The inflammatory response is strongly correlated with the onset and progress of renal damage in diabetic subjects (Navarro-Gonzalez et al., 2011), but its precise role in the progression of DN is still obscure.

In recent years, the modification of gut microbiota (GM) and activation of inflammation pathways have been implicated in the development of DN, with the conception of a gut–kidney axis being proposed, although the causative relationship remains to be elucidated (Yang et al., 2018). Indeed, the GM has been identified as a promoter or mediator of systemic inflammation. Dysbiosis, intestinal barrier dysfunction, and bacterial translocation can cause inflammation in chronic kidney disease (CKD) (Andersen et al., 2017). Therefore, the onset of early kidney complications associated with diabetes may be prevented by modulating the GM.

Resveratrol, a stilbene polyphenolic compound, is widely present in grapes, mulberries, red wines and peanut skins, which has been studied in various animal model of human diseases, such as diabetes and coronary heart disease (Baur et al., 2006; Singh et al., 2015). Resveratrol can inhibit oxidative stress and has anti-inflammatory and anti-apoptotic properties (Madeo et al., 2019). Increased research attention in the field of DN has focused on its potential value in protecting kidney damage. Our previous studies in diabetic mouse models have indicated that orally administered resveratrol can have nephroprotective effects (Ding et al., 2010; Huang et al., 2017). However, the pathophysiologic mechanisms underlying the effects of resveratrol on DN are still obscure. Owing to the low bioavailability of resveratrol, it has been postulated that a mechanism of resveratrol efficacy is mediated through its interaction with the gut. In fact, several mechanisms have been proposed by which resveratrol can exert its health effects through modulating the composition of the gut microbiota (Bird et al., 2017; Komaroff, 2017). It has been reported that ingestion of resveratrol induced changes in the gut microbiome composition and improvements in glucose homeostasis of obese and diabetic mice (Sung et al., 2017b; Chaplin et al., 2018). However, the effect of resveratrol on the GM and the development of DN remains largely unclear.

In the present study we investigate whether resveratrol exerts anti-DN effects by modulating the composition of gut microbiome, intestinal barrier dysfunction, and the inflammatory response.

## Materials and Methods

### Animal Experiments

The Male C57BL/KsJ diabetic db/db and db/m mice were used in this study. Db/db mice are a genetic model of early stage type 2 DN with hyperglycemia and urinary albumin excretion enhancement, while db/m mice served as the control (Madeo et al., 2019). The mice were housed in well-ventilated plastic cages with stainless steel grid tops at 22 ± 2°C with a 12 h light/dark cycle. At 8 weeks of age, the mice were randomly assigned into four groups (db/m, n=6; db/m+resveratrol, n=7; db/db, n=6; and db/db+resveratrol, n=7). Multiple cages for each treatment group were set up housing two to three mice per cage. The db/m+resveratrol and db/db+resveratrol mice were treated with resveratrol by oral gavage at a dose of 10 mg/kg/day for 12 weeks. The db/m and db/db groups received an equivalent amount of saline by oral gavage for the same period. The dosage was adjusted for body weight changes every week for the entire study period. During the experiment, all mice was received the same standard chow. Fasting blood sugar level (FBS) was measured by a glucometer (Abbot, Alameda, CA, USA) every 2 weeks in all animals. A BP-2000 blood pressure analysis system (Visitech Systems, Apex, NC) was used to assess systolic blood pressure (SBP) by tail cuff plethysmography in conscious mice. SBP was measured 10 times per day for 4 consecutive days, and a mean value was calculated for each mouse. Urine was collected from the metabolic cage after housing individual mouse for 24-h and was stored at -80°C until analysis. At the end of the experiment, blood samples were collected by the retro-orbital plexus for biochemical assays. Kidneys, intestine and fecal samples were also collected.All animal experiments were conducted in accordance with the committee guidelines of the Nanjing Medical University and approved by the Institutional Animal Care and Use Committee of Nanjing Medical University, No.14030134.

### Biochemical Analyses and Cytokine Measurements

The serum levels of creatinine (SCr) and blood urea nitrogen (BUN) were assessed using an automated biochemical analyzer (Hitachi 7060, Tokyo, Japan). In order to evaluate the level of inflammation in mice, ELISA was used to measure serum levels of endotoxin lipopolysaccharide (LPS), pro-inflammatory cytokines interleukin(IL)-6 and interleukin (IL)-1β, tumor necrosis factor (TNF)-α, and interferon (IFN)-γ), according to the manufacturer’s instructions (ELISA, Nanjing Jiancheng Bioengineering Institute, Nanjing, China). Twenty-four-hour microalbuminuria was also assessed by ELISA, according to the manufacturer’s instructions.

### Histopathological Analysis and Immunohistochemistry

Kidney and small intestine tissue samples were sent to the Pathology laboratory of The Second Affiliated Hospital of Nanjing Medical University for H&E or PAS staining. For immunohistochemical staining, sections were deparaffinized, heated in citrate-buffered solution (pH 6.0) for antigen retrieval, and incubated with anti-mouse primary antibody against IFN-γ (Abcam 175878, USA), TNF-α (Abcam ab6671, USA), ZO-1 protein (Invitrogen, 61-7300 Shanghai, China), or Claudin-1 protein (Invitrogen, 32-5600 Shanghai, China) overnight at 4°C. Sections were incubated with biotinylated secondary antibodies, followed by adding diaminobenzidine tetrahydrochloride substrate to visualize the antibody staining. A pathologist reviewed kidney histology and staining in a blinded manner. Histological scoring was performed by measuring intestine damage as previously described (Maslowski et al., 2009).

### Intestinal Permeability

Intestinal permeability was determined by measuring plasma levels of fluoresceinisothiocyanate conjugated-dextran (FD-4). Mice were orally gavaged with 0.5 ml of FD-4 (22 mg/ml, molecular mass 4.4 kDa) 5 h prior to euthanasia. Plasma was harvested at the time of euthanasia and 50μl of plasma was diluted with an equivalent volume of phosphate-buffered saline (PBS pH 7.4), and the concentration of FD-4 was measured spectrofluorometrically (NanoDrop 3300, Thermo Scientific, Wilmington, DE) as described previously (Ding et al., 2010). Serially diluted samples were used as standards, and all samples were analyzed in triplicate.

### Quantitative Real-Time PCR

Quantitative PCR was performed using the StepOne Plus Real-Time PCR system (Applied Biosystems, Thermo Fisher Scientific, Inc). Primer sequences for the targeted mouse genes were as follows:

IL-6, forward: 5′-ACAACCACGGCCTTCCCTACTT-3′, reverse: 5′-CACGATTTCCCAGAGAACATGTG-3′;

IL-1, forward: 5′-TCTTTGAAGTTGACGGACCC-3′, reverse: 5′- TGAGTGATACTGCCTGCCTG-3′;

TNF-α, forward: 5′-GAAGTTCCCAAATGGCCTCC-3′ reverse: 5′-GTGAGGGTCTGGGCCATAGA-3′;

IFN-γ, forward: 5′-TGTCATCGAATCGCACCTGA-3′ reverse: 5′-TCAGCACCGACTCCTTTTCC-3′;

Total RNA was isolated from mice kidneys with TRIzol (Invitrogen, Carlsbad, CA) reagent according to the manufacturer’s instructions. Gene expression was calculated as △CT using GAPDH as a reference, and was expressed relative to the control group normalized to a value of 1.

### Fecal Microbiota Transplantation

Fresh fecal matter was collected from mice treated with resveratrol or control db/m mice as the donor of fecal microbiota transplantation (FMT). Meanwhile, the fresh transplant material was prepared on the same day of FMT within 10 min before oral gavage to prevent changes in bacterial composition. A separate cohort of male db/db mice (8 weeks old, n=16) underwent adaptive feeding for 1 week, then received a cocktail of antibiotics in their drinking water for three consecutive days (vancomycin, 100 mg/kg; neomycin sulfate, 200 mg/kg; metronidazole, 200 mg/kg; and ampicillin, 200 mg/kg). After completion of the antibiotics treatment, mice were fasted overnight (day 0) and the fasting blood glucose (FBS) and the systolic blood pressure (SBP) were measured. Then mice were randomly assigned to two groups, one group of mice received FMT by using a fecal suspension from donor db/m mice (C, control-FMT, n = 8), the other group received FMT by using a fecal suspension from donor db/m+resveratrol mice (R, resveratrol-FMT, n = 8) by oral gavage (200 μl; each FMT dose was prepared from an average weight of 40 mg of fecal matter, equivalent to 2 fresh fecal pellets from the donor). FMT of db/db mice were repeated six additional times on consecutive days (without fasting) for a total of seven FMT per mouse. During this time, all recipient mice continued to have access to standard chow. The changes in body weight and blood sugar were recorded daily for four weeks after FMT treatment. After treatment, the recipient mice were euthanized and blood, intestines and kidneys were collected as described above.

### Data Collection, 16S rRNA Sequencing, and Quality Control

The DNA was extracted from 200 mg samples using the QIAamp DNA Stool Mini Kit (QIAGEN, Hilden, Germany) following the manufacturer’s instructions. Isolated DNA was stored at −20˚C for further study. The V4–V5 region of 16S rRNA gene was amplified in each sample using universal primers 515F(5’-GTGCCAGCMGCCGCGGTAA-3’) and 926R (5’-CCGTCAATTCMTTTGAGTTT-3’). The PCR products from different samples were indexed and mixed at equal ratios for sequencing by 2*300 bp paired-end sequencing on the MiSeq platform using MiSeq v4 Reagent Kit (Illumina) at TinyGen Bio-Tech (Shanghai) Co., Ltd.

### Gut Microbiota Analysis

The raw fastq files were demultiplexed and pair-end reads for all samples were processed through Trimmomatic (version 0.35) to remove low quality base pairs using the following parameters: SLIDINGWINDOW: 50:20 MINLEN: 50. Trimmed reads were then further merged using the FLASH program (version 1.2.11) with default parameters. The low quality contigs were removed based on screen.seqs command using the following filtering parameters, maxambig=0, minlength = 200, maxlength =580, maxhomop= 8. After removing low-quality reads a total of 733,481 high-quality reads remained (range from 17,557 to 38,441 reads per sample). The demultiplexed reads were clustered into 510 operational taxonomic units (OTUs) at 97% sequence identity using the UPARSE pipeline, which were then assigned for taxonomy against the Silva 119 database by the “classify.seqs” command in mothur with a confidence score ≥ 0.8. OTU taxonomies (from Phylum to Species) were determined based on NCBI. Based on the results of the optimized sequence OTUs, clustering analysis, a variety of diversity index analysis (alpha diversity analysis in the sample) and detection of sequencing depth was performed. Statistical analysis of community structure at each classification level and beta diversity analysis between samples were also performed.

The 16S sequences were analyzed using a combination of software mothur (version 1.33.3),UPARSE (usearch version v8.1.1756, http://drive5.com/uparse/), and R (version 3.6.1). A principal component analysis (PCA) using the package ade4 implemented in R software (R 3.6.1) was performed. The α-diversity of gut microbiota and the community composition of the samples was drawn using R. The statistical significance of differences in bacterial composition among the different samples was assessed by either the Wilcox or the Kruskal-Wallis test.

### Data Availability

All the 16S rRNA sequencing data required to assess the conclusions of this research are available without restrictions from the Sequence Read Achieve (SRA) at the National Center for Biotechnology Information (NCBI) under BioProject accession PRJNA628114.

### Statistical Analysis

Statistical significance of the differences between the groups of mice was assessed using R 3.6.1 and GraphPad Prism software v 5.0 (GraphPad Software, San Diego, CA, USA). PCA were performed using SIMCA-P software to cluster the sample plots across groups. Differential abundances of genera were tested by one-way analysis of variance (ANOVA) and non-parametric tests, including the Wilcoxon rank-sum test and Mann–Whitney U test. Data are presented as the means ± SEM for the indicated number of independently performed experiments, and p values <0.05 were considered statistically significant.

## Results

### Resveratrol Prevents the Progression of Diabetic Nephropathy in db/db Mice

**Figure 1:**
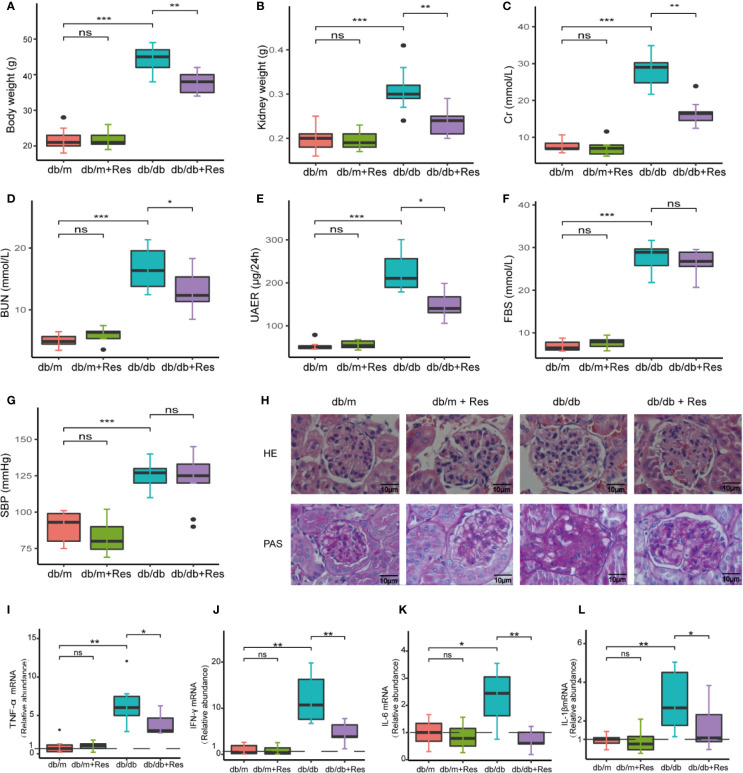
Resveratrol relieves a series of indicators of DN and improves glomerular histological abnormalities in db/db mice. **(A–G)** Quantitative analysis of body weight, kidney weight, BUN, Cr, UAER FBS and SBP in db/db and db/m control mice treated with either saline or resveratrol. **(H)** Representative photomicrographs of HE-stained and PAS-stained kidney sections (magnification: 400×). **(I–L)** Kidney mRNA levels of TNF-α, IFN-γ, IL-6, IL-1β relative to GAPDH Bar graphs show densitometry analysis of specific bands expressed and was expressed relative to the control group normalized to a value of 1. Data is representative of multiple mice per group (db/m: n=6, db/m+Res: n=7, db/db: n=6, db/db+Res: n=7). Data are presented as mean ± SEM. ***p<0.001; **p<0.01; *p<0.05; and ns: not significant.

To investigate the potential therapeutic effect of resveratrol on DN in a db/db diabetic mouse model. As expected, body and kidney weights of db/db mice were significantly higher than control db/m mice (**Figures 1A, B**). In comparison to saline-treated db/db mice, resveratrol treatment of db/db mice significantly reduced body (**Figure 1A**) and kidney (**Figure 1B**) weights and significantly decreased levels of serum creatinine (Cr) (**Figure 1C**), blood urea nitrogen (BUN) (**Figure 1D**) and urine 24-h microalbuminuria (UAER) (**Figure 1E**). The fasting blood sugar (FBS) (**Figure 1F**) and the systolic blood pressure (SBP) (**Figure 1G**) were not significantly affected by resveratrol treatment in the db/db mice group. In contrast, resveratrol-treatment did not affect the body and kidney weights and the biochemical parameters in db/m mice (**Figures 1A–G**).

Next, we examined whether resveratrol administration influenced the renal morphology in db/db mice. Renal tissues of db/db mice (**Figure 1H**) showed a series of indicators of DN, including glomerulus hypertrophy, capillary basement membrane thickening, and mesangial matrix expansion, as compared to db/m mice (**Figure 1H**). After resveratrol treatment, glomerular lesion formation was remarkably alleviated (**Figure 1H**). Moreover, resveratrol treatment significantly decreased the expression of mice (**Figures 1I–L**). Together, these results indicate that resveratrol ameliorates the DN-associated functional abnormalities, renal morphological changes, and renal inflammatory markers in db/db mice.

### Resveratrol Improves Intestinal Barrier Function and Ameliorates Intestinal Permeability and Inflammation

**Figure 2:**
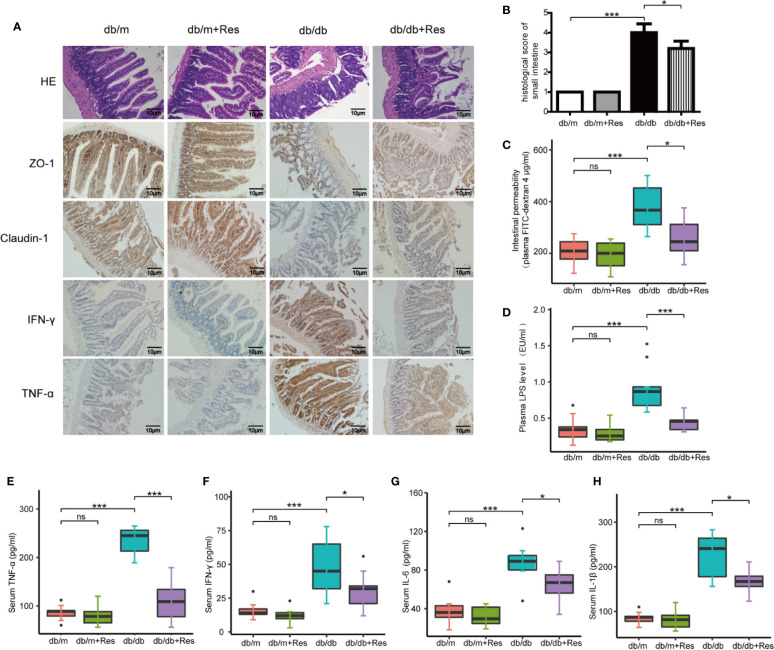
Resveratrol improves intestinal barrier function and ameliorates intestinal permeability and inflammation. **(A)** Representative photomicrographs of HE-stained small intestine sections and photomicrographs of immunofluorescence for ZO-1, Claudlin-1, IFN-γ, and TNF-α in db/db and db/m control mice treated with either saline or resveratrol (Magnification: 200×). **(B)** Histological score of small intestine damage in db/db and db/m control mice treated with either saline or resveratrol. **(C)** Plasma FITC-dextran 4 (DX, 4,000 molecular weight) levels after oral challenge in db/db and db/m control mice treated with either saline or resveratrol. **(D)** The levels of plasma endotoxin (LPS), EU: endotoxin units, in db/db and db/m control mice treated with either saline or resveratrol. **(E–H)** The levels of serum TNF-α, IFN-γ, IL-6, IL-1β in db/db and db/m control mice treated with either saline or resveratrol. Data is representative of multiple mice per group: db/m: n=6, db/m+Res: n=7, db/db: n=6, db/db+Res: n=7). Data are presented as the mean ± SEM. ***p<0.001; *p<0.05; and ns: not significant.

The progression of kidney disease could be mediated through an impaired gut-kidney axis. We therefore examined the effect of resveratrol on intestinal permeability, barrier function and intestinal inflammation in db/db and db/m mice. The intestinal histopathology was evaluated by H&E staining using a scoring system that takes into account mucosal ulceration and depth of injury (Chaplin et al., 2018). The histological score was significantly higher in the db/db mice group compared to the db/m mice group, while the histological score was significantly lower in the resveratrol-treated db/db mice than in saline treated db/db mice (**Figures 2A, B**). Intestinal permeability was monitored by measuring the plasma levels of 4 kDa FITC-dextran 4. As shown in **Figure 2C**, a higher level of 4 kDa FITC-dextran 4 was observed in db/db mice compared to db/m mice, which showed that diabetic mice had an increased gut leakiness. Interestingly, resveratrol treatment significantly decreased the level of 4 kDa FITC-dextran in db/db mice, indicating that resveratrol ameliorates intestinal permeability. Furthermore, we confirmed the impaired gut barrier in db/db mice by immunostaining for tight-junction proteins ZO-1 and claudin-1. Decreased expression of ZO-1 and claudin-1 was detected in db/db mice compared to db/m mice. Importantly, resveratrol treatment substantially recovered ZO-1 and claudin-1 protein expression in db/db mice (**Figure 2A**). In contrast, the intestinal damage and permeability were not significantly different between resveratrol-treated and untreated db/m mice. To further demonstrate the impact of resveratrol on intestinal inflammation, immunostaining showed that the levels of IFN-γ and TNF-α are significantly higher in db/db mice when compared to db/m mice (**Figure 2A**). Resveratrol treatment significantly decreased the levels of IFN-γ and TNF-α in the small intestine of db/db mice (**Figure 2A**). We also confirmed that resveratrol decreased plasma levels of LPS, IFN-γ, TNF-α, and IL-6 in db/db mice (**Figures 2D–H**). Together these results suggest that resveratrol can protect against gut barrier dysfunction, mucosal inflammation and endotoxemia.

### Effects of Resveratrol on Gut Microbial Structure in db/m Mice and db/db Mice

**Figure 3:**
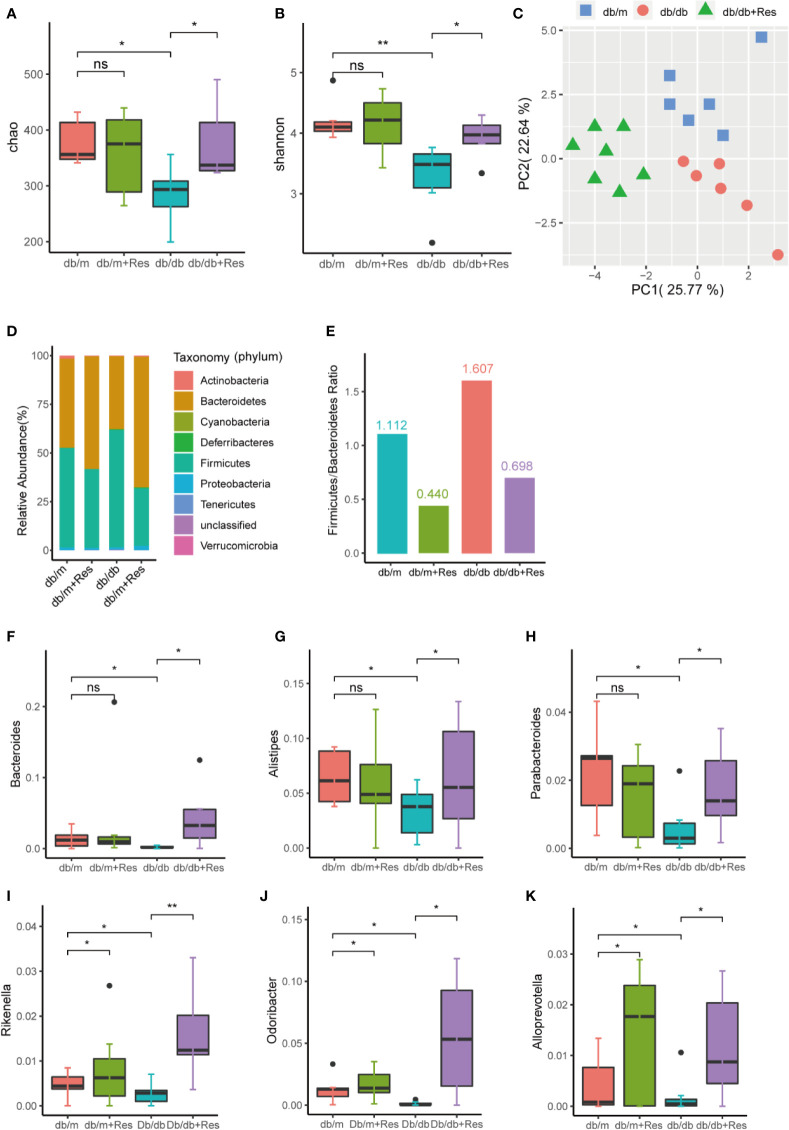
Effects of resveratrol on gut microbiota in db/m and db/db mice. Male db/db and db/m control mice were treated with either saline or resveratrol: db/m(n=6), db/m+resveratrol (db/m+Res,n=7), db/db(n=6, db/db+resveratrol(db/db+Res,n=7). Microbiome composition was determined by 16S rRNA gene sequencing. **(A, B)** Chao and Shannon alpha-diversity index in db/m groups, db/m+Res, db/db, db/db+Res mice. **(C)** Principal-component analysis (PCA) of fecal microbiome in db/m mice, db/db and db/db+Res mice (db/m: blue square; db/db: red dots;db/db+Res: green triangle). **(D)** Gut microbe relative abundance at the phylum level in db/m, db/m+Res, db/db, and db/db+Res mice. **(E)** *Firmicutes*/*Bacteroidetes* ratio in db/m, db/m+Res, db/db, and db/db+Res mice. **(F–K)** Relative species abundance at the genus level for *Bacteroides, Alistipes, Parabacteroides, Rikenella, Odoribacter and Alloprevotella* in db/m, db/m+Res, db/db, and db/db+Res mice. Data are represented as the mean ± SEM. **p<0.01; *p<0.05; and ns: not significant.

To elucidate the mechanism of the renoprotective effect of resveratrol treatment, we investigated the impact of resveratrol on the gut microbiota in db/db mice. 16S rDNA sequencing was used to assess changes in the fecal microbiota of db/m and db/db mice with or without resveratrol treatment. From 26 samples, after removing low-quality reads total 733,481 high-quality reads remained (range from 17,557 to 38,441 reads per sample) and 510 OTUs were obtained.The α-diversity was significantly lower in db/db mice compared to db/m mice (**Figures 3A, B**, **Table S1**), whereas resveratrol treatment increased bacterial richness and α-diversity in db/db mice, but not in db/m mice (**Figures 3A, B**). Principal-component analysis (PCA) of 16S rRNA sequence data of feces contents showed a clear separation based on community structure between db/db and db/m mice (**Figure 3C**), and between saline- and resveratrol-treated db/db mice (**Figure 3C**). The two most abundant phyla across the four treatment groups were *Firmicutes* and *Bacteroidetes*. Resveratrol treatment decreased the relative abundance of *Firmicutes* and increased abundance of *Bacteroidetes* in both db/db and db/m mice (**Figure 3D**), which was further quantified by calculating the *Firmicutes*/*Bacteroidetes* (F/B) ratio (**Figure 3E**). The overall image of bacterial composition at the species level in four groups showed in **Figure S1**. An increased F/B ratio has been associated with diabetes and obesity. However, the abundance of *Proteobacteria* was low, with no significant difference between groups. At the genus level, we identified six OTUs that were particularly responsive to the interaction between mice genotype and resveratrol treatment. Indeed, as shown in **Figures 3F–K**, the fecal microbiota of resveratrol-treated db/db mice was enriched for the following six genera: *Bacteroides*, *Alistipes*, *Rikenella*, *Odoribacter*, *Parabacteroides*, and *Alloprevotella* when compared to the db/db mice group (**Table S2**). Importantly, *Bacteroides*, *Alistipes* and *Parabacteroides* exhibit anti-inflammatory properties and were markedly increased in the resveratrol-treated db/db mice group, with an increase of 21-fold, 2-fold and 2.8-fold respectively (**Figures 3F–H**) (Chung et al., 2012). These results suggest that the gut microbiota composition in db/db mice was significantly altered compared to db/m mice and that resveratrol could at least partially rescue this change in db/db mice.

### Transplantation of the Resveratrol-Modified Fecal Microbial Community Is Sufficient to Improve Renal Function of db/db Mice

**Figure 4:**
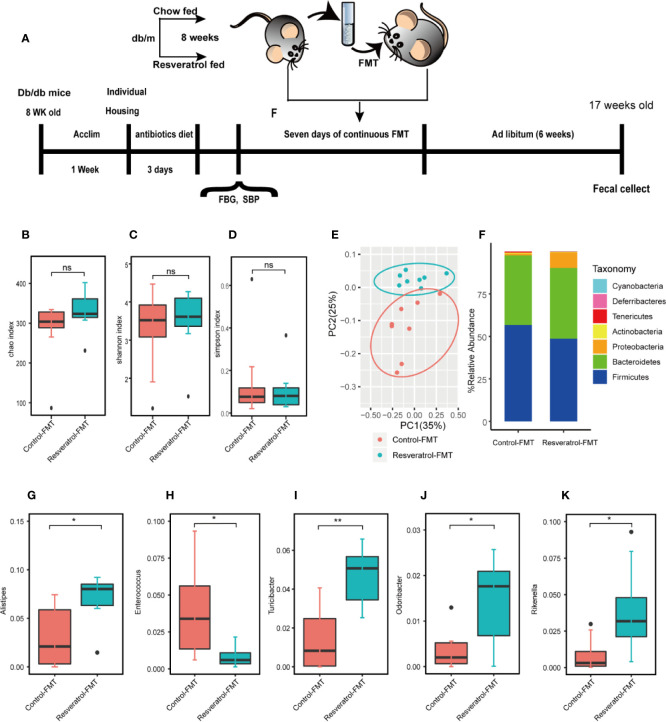
Gut microbiome composition of db/db fecal microbiota transplantation recipients from donor db/m mice fed a resveratrol or control diet. **(A)** Male db/db mice (8 weeks old, n=16) were acclimated for one week, then treated with an antibiotics cocktail for three days. Microbiome depleted mice were randomly assigned to one of two groups receiving Fecal Matter Transplantations (FMTs) from either db/m mice fed a control chow diet (C, control-FMT, n = 8) or db/m mice fed a resveratrol diet (R, resveratrol-FMT, n = 8) for seven consecutive days. After the completion of antibiotics treatment before the first FMT, Fasting Blood Sugar (FBS) and Systolic Blood Pressure (SBP) were measured. Following an overnight fast (day 0), fecal matter from db/m mice was transplanted *via* oral gavage once a day for 7 consecutive days. All mice receiving FMT had access to standard chow at all times during and after FMT. Fecal samples were collected and SBP, FBG, CR, BUN, UREA levels were measured four weeks after completion of FMT. Tissues were collected after a 5–6 h fast on day 4 in the fourth week. **(B–D)** Chao, Shannon and Simpson alpha-diversity index in db/db mice following control-FMT (n = 8) or resveratrol-FMT (n = 8). **(E)** Principal-component analysis (PCA) of 16S rRNA fecal microbiome of db/db mice following control-FMT (n = 8) or resveratrol-FMT (n = 8) (control-FMT: red dots; resveratrol-FMT: blue dots). **(F)** Gut microbiome relative abundance at the phylum level in db/db mice following control-FMT (n = 8) or resveratrol-FMT (n = 8). **(G–K)** Relative species abundance at the genus level for *Alistipes, Enterocuccus, Turicbacter, Odoribacter and Rikenella* in control-FMT (n = 8) or resveratrol-FMT (n = 8) mice. **p<0.01; *p<0.05; and ns: not significant.

**Figure 5:**
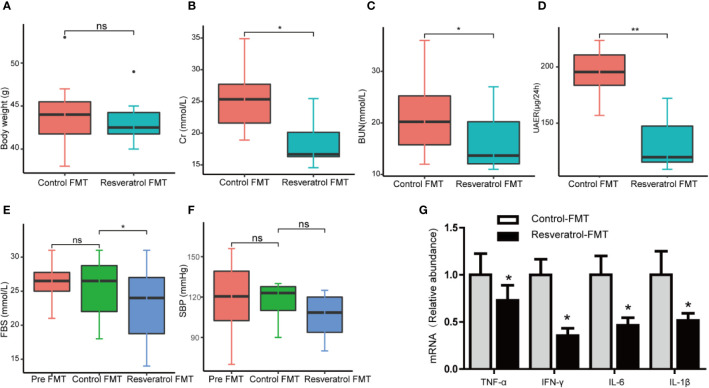
Fecal microbiota transplantation from db/m mice fed a resveratrol diet to db/db recipient mice improved renal function. **(A–F)** Quantitative analysis of body weight, Cr, BUN, 24h UAER, FBS, and SBP in control-FMT and resveratrol-FMT mice. **(G)** Representative kidney mRNA levels of TNF-α, IFN-γ, IL-6, and IL-1β by RT-PCR analysis in control-FMT (n=8) and resveratrol-FMT (n=8) mice. Data are represented as the mean ± SEM **p<0.01; *p<0.05; and ns: not significant.

In order to confirm whether resveratrol-mediated changes in the intestinal microbiota contribute to improvement of renal function, fecal slurries collected from saline- (control-FMT) or resveratrol-treated (resveratrol-FMT) db/m mice were transplanted into single-housed and antibiotics-pretreated db/db mouse by oral gavage daily for 7 days (**Figure 4A**). The use of antibiotics treatment is to remove the original gut microbiota of db/db mice, and to construct a relatively sterile environment to ensure the effect of FMT. After FMT treatment, mice were followed up for 4 weeks (**Figure 4A**). 16S rRNA sequencing analysis of fecal samples from FMT recipient mice showed that the α-diversity of the microbiota in resveratrol-FMT recipient mice was not significantly different compared to control-FMT db/db mice, as reflected by the Chao, Shannon and Simpson indexes (**Figures 4B–D**, **Table S3**). On the other hand, 16S rRNA sequencing analysis verified that resveratrol-FMT reconstituted the gut microbiome of db/db mice, principle component analysis (PCA) differentiated resveratrol-FMT mice from the control-FMT db/db mice (**Figure 4E**). At the phylum level, Firmicutes and Bacteroidetes dominated the gut microbiome of both groups similar to the gut microbiome composition of db/m and db/db mice (**Figure 4F**). Db/db mice receiving resveratrol-FMT were characterized by decreased proportions of *Firmicutes, Tenericutes* and *Deferribacteres* as well as increased proportions of Proteobacteria compared to levels in control-FMT db/db mice. At the genus level, resveratrol-FMT db/db mice exhibited increased abundance levels of *Alistipes, Turicibacter, Odoribacter* and *Rikenella* genus and lower abundance of Enterococci compared to control-FMT mice (**Figures 4G–K**, **Table S4**), which is similar to our results obtained with oral resveratrol treatment in db/db mice. These findings were also confirmed by heatmap analysis of the bacterial taxons, which could significantly separate the resveratrol-FMT recipient mice from the control-FMT db/db mice (**Figure S2**). These results indicated that resveratrol-FMT could significantly influence the structure and composition of the gut microbiome. We observed no difference in body weight between resveratrol-FMT and control-FMT db/db mice (**Figure 5A**). Importantly, in comparison to the control-FMT mice, the resveratrol-FMT mice showed significantly decreased urine 24-h microalbuminuria (UAER), serum creatinine (Cr), blood urea nitrogen (BUN) and fasting blood sugar (FBS) levels (**Figures 5B–E**). The Systolic blood pressure (SBP) was lower in the resveratrol-FMT mice, but was not significantly different compared to control-FMT mice (**Figure 5F**). Finally, we discovered that resveratrol-FMT mice showed significantly lower kidney mRNA levels of TNF-α, IFN-γ, IL-6, and IL-1β compared to control-FMT recipients (**Figure 5H**), suggesting that reduced inflammation by resveratrol-modified fecal microbial community may be a key mechanism for protecting the renal function of DN.

### Transplantation of the Resveratrol-Modified Fecal Microbial Community Improved Intestinal Characteristics and Decreased Inflammatory Response

**Figure 6:**
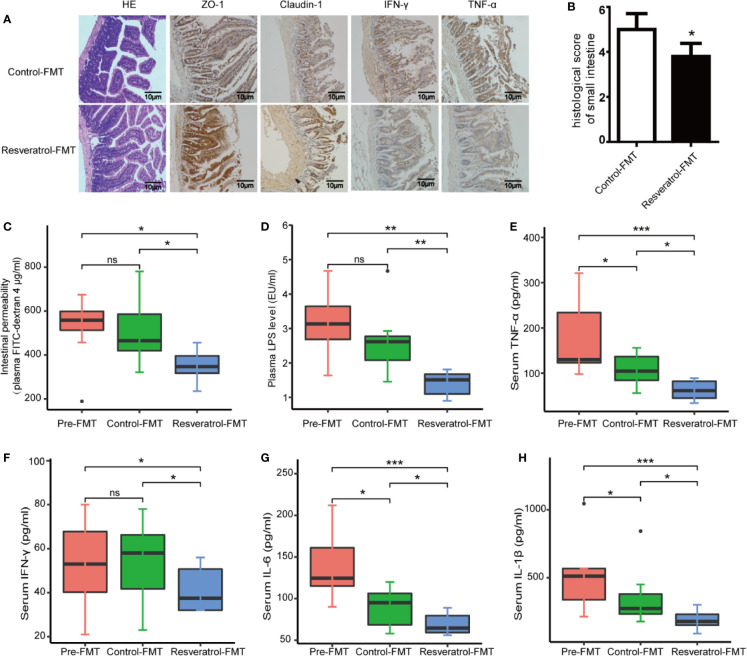
Transplantation of the resveratrol-modified fecal microbial community improved intestinal characteristics and decreased inflammatory response in db/db mice. **(A)** Representative photomicrographs of H&E-stained small intestine sections and immunofluorescence for ZO-1, Claudlin-1, IFN-γ and TNF-α (magnification: 200×). **(B)** Representative histological scoring of small intestine damage. **(C)** Plasma FITC-dextran 4 (DX, 4,000 molecular weight) levels after oral challenge in control post-FMT (n=8) or resveratrol post-FMT (n=8) mice. **(D)** Plasma levels of LPS (endotoxin units/ml). **(E–H)** Serum levels of TNF-α, IFN-γ, IL-6, and IL-1β by ELISA in control post-FMT (n=8) or resveratrol post-FMT (n=8). ***p<0.001; **p<0.01; *p<0.05; and ns: not significant.

Next we analyzed intestinal characteristics, gut inflammation and serum inflammatory response between control-FMT, resveratrol-FMT and pre-FMT db/db mice. First, the intestinal histopathology was evaluated by H&E staining. Resveratrol-FMT mice had a lower histological score for mucosal ulceration and depth in the small intestine compared to control-FMT recipients (**Figures 6A, B**). Intestinal permeability measured by the plasma levels of orally administered 4 kDa FITC-dextran 4 showed decreased permeability in Resveratrol-FMT mice compared to control-FMT and pre-FMT mice (**Figure 6C**), which was further confirmed by immunohistochemical staining using ZO-1 and claudin-1 of small intestine samples (**Figure 6A**). To further demonstrate the impact of resveratrol-FMT on intestinal inflammation, immunostaining showed that the levels of IFN-γ and TNF-α were lower in resveratrol-FMT mice compared to control-FMT mice (**Figure 6A**). Similarly, when compared to control-FMT recipients or pre-FMT mice, resveratrol-FMT recipients showed significantly lower levels of plasma LPS and serum IFN-γ, TNF-α, IL-6, and IL-1β demonstrating that transplantation of the resveratrol-modified fecal microbial community also reduced systemic inflammation (**Figures 6E–H**). These results suggest that the anti-DN effects of resveratrol are at least partly mediated through modulation of the gut microbiota.

## Discussion

Increasing evidence supports the causative role of gut microbiota in DN and CKD development and progression. The underlying mechanisms involve diabetes induction, inflammatory response activation, and metabolism deregulation (Knauf et al., 2019). Our present study using an animal model of diabetic nephropathy demonstrated that the dysbiosis mediated-inflammatory response is involved in the development of diabetes-associated kidney disease. In addition, we showed for the first time that the protective effect of resveratrol on DN is mediated *via* modulation of the intestinal microbiota and related gut-kidney axis activation, counteracting the inflammasome initiation response as well as renal dysfunction.

In particular, the increased pro-inflammation factors play a critical role in the progression of renal dysfunction in DN. Resveratrol, administered orally, relieved a series of indicators of diabetic nephropathy, such as the level of serum BUN, CR, 24-hour urine microalbumin, and the renal inflammation response. This is consistent with previous reports, which showed that resveratrol ameliorated type 2 diabetes-induced renal failure diseases, through its antioxidant, anti-inflammatory mechanisms (Palsamy and Subramanian, 2011; Li et al., 2018).

Chronic low-grade inflammation is a pathophysiological feature of diabetes. Intestinal barrier dysfunction and increased intestinal permeability promotes a chronic blood inflammation response, resulting in increased exposure of host tissues, including the kidneys, to endotoxins (Sabatino et al., 2017). In this study, we found higher intestinal permeability in db/db mice compared to db/m mice, which showed that diabetes in mice promoted gut leakiness. Importantly, we first showed that resveratrol treatment decreased intestinal permeability in db/db mice. This is similar to previous reports, which showed that an increase in gut permeability caused by the common mycotoxin deoxynivaenol could be arrested by resveratrol (Ling et al., 2016). The increase in intestinal permeability was also associated with decreased expression of tight-junction, and increased small intestinal lesions (Etxeberria et al., 2015). A major concern with intestinal permeability is the increased passage of LPS from the gut lumen to the plasma. Indeed, LPS was increased in the blood of diabetic individuals, and it has been implicated in induction of systemic inflammation, insulin resistance, renal dysfunction, and uremic toxins in DN (Mahmoodpoor et al., 2017; Koppe et al., 2018). However, whether alteration of the intestinal environment by resveratrol in the small intestine is associated with amelioration of the progression in DN is not known. Here, we show that addition of resveratrol prevents the increase in both the level of plasma LPS and the levels of serum inflammation markers. Our findings provide important information on how resveratrol protects against gut barrier dysfunction, with consequences for inflammatory-mediated endotoxemia in db/db mice. Previous studies have suggested that resveratrol exerts protective effect on gut barrier function by inhibiting MAPK, NF-B, and JAK/STAT pathways, thus suppressing the generation of proinflammatory. Resveratrol also protected inflammatory bowel disease and improved gut barrier function by downregulating the expression of HIF-1, mTOR, and STAT3 pathway (Nunes et al., 2018).

There is considerable evidence to show that the role of the gut microbiota on intestinal barrier dysfunction and bacterial translocation is to cause and perpetuate inflammation (Thevaranjan et al., 2017). Furthermore, animal studies in which the gut microbiota is manipulated, as well as observational studies in patients with CKD, have provided considerable evidence that dysbiosis contributes to the process of CKD (Andersen et al., 2017). Although previous reports have found that resveratrol modulated the composition of the gut microbiota, suggesting that the microbiome plays a critical role in the progression of diabetes and obesity (Sung et al., 2017b; Wang et al., 2020), the beneficial role of resveratrol on the microbiota specifically in DN is unclear.

In this study, we found that the ratio of *Firmicutes*/*Bacteroidetes* in the db/db mice group was increased compared with that in the control group, while resveratrol restored the ratio. Previous studies have also shown that the ratio of *Firmicutes*/*Bacteroidetes* plays an important role in metabolic diseases, such as diabetes and obesity (Abdallah et al., 2011). In addition, the resveratrol-treated group showed an increased abundance of *Bacteroides*, *Alistipes*, *Rikenella*, *Odoribacter*, *Parabacteroides*, and *Alloprevotella* genera when compared to the db/db mice group. In particular, *Bacteroides*, *Alistipes*, and *Parabacteroides* have been significantly associated with anti-inflammatory factors (Troy and Kasper, 2010). Similarly, several studies in mice showed that resveratrol caused gut microbiota remodeling with an increase in the relative abundance of *Bacteroides* and *Parabacteroides*, which attenuated trimethylamine-N-oxide (TMAO) induced atherosclerosis and improved exercise performance and skeletal muscle oxidative capacity in heart failure (Chen et al., 2016; Sung et al., 2017a).Within db/m mice, we observed a significant increase in the abundance of Alistipes, Odoribacter and Alloprevotella genera changes very similar to those in the resveratrol-treated db/db mice. The increased abundance of the *Alloprevotella* in the db/db+resveratrol has previously been linked to beneficial intestinal health and to aberrant production of metabolic endotoxin (Larsen et al., 2010). Thus, the anti-inflammatory effect of resveratrol may be seen to modulate the bacterial composition of these candidates in db/db mice. The *Odoribacters*, which are known for their butyrate-producing capabilities in the human gut, were increased by the resveratrol treatment. *Odoribacters* are also capable of tryptophan metabolism and glycolysis processing (Gomez-Arango et al., 2016). Therefore, we propose that resveratrol may promote the growth of bacteria involved in glycolysis in db/db mice, resulting in the improvement of glucose homeostasis.

Diabetes and CKD are known to be associated with a significant decrease in microbiota diversity, as well as with altered barrier function and increased permeability of the epithelium (Evenepoel et al., 2017). By contrast, dysbiosis, intestinal barrier dysfunction, and the perpetuation of inflammation are ascribed to renal dysfunction in DN (Andersen et al., 2017). In the current study, and to confirm a causal relationship between changes in the fecal microbiome and the observed renal dysfunction, we transplanted the resveratrol-altered gut microbiota from db/m mice to db/db mice. The results of our fecal microbiota transplantation experiments supported our hypothesis that the resveratrol-mediated microbiota is a driving force in improving renal function, improving glucose homeostasis, restoring gut permeability, and reducing inflammatory markers. Within the fecal microbiota analysis, our findings showed that transplantation of the resveratrol-modified fecal microbiota from donor mice increased the abundance of Proteobacteria and decreased the abundance of Firmicutes. This trend of Firmicutes level is similar to the microbiota change in the resveratrol-mediated db/db mice. Similarly, at the genus level, the increased abundances of Alistipes, Odoribacter and Rikenella in resveratrol-FMT mice are consistent with resveratrol administration in db/db mice. Importantly, resveratrol-FMT significantly increased the abundance of *Turicibacter* in db/db mice, which is inconsistent report that resveratrol decreased the abundance of *Turicibacter* in obese mice (Sung et al., 2017b). However, *Turicibacter* can modulate inflammatory responses and exert an anti-inflammatory effect in inflammatory bowel disease (IBD) (Loh and Blaut, 2012), contributing ultimately to metabolic dysfunction. We also observed a significant change in fecal abundance of *Odoribacter* in resveratrol-FMT mice, which may in part be the reason for the resveratrol-FMT mice having decreased glucose levels (Gomez-Arango et al., 2016). In previous studies, the number of aerobic bacteria, such as *Enterobacteria* and *Enterococci*, was dramatically higher in hemodialysis patients, resulting in an increase in some uremic toxins (Perna et al., 2019). In the current study, the transplantation of resveratrol-altered gut microbiota significantly lowered the abundance in the *Enterococcus* genus, which previously has been linked to beneficial anti-inflammatory effects in targeted intervention studies, both in rodents and in humans (Daillere et al., 2016). Moreover, we found that the abundance of the *Parabacteroides* genera in resveratrol-FMT mice were higher than this in the control-FMT mice, which were consistent with previous studies showing that resveratrol increased *Parabacteroides* genera (Sung et al., 2017b; Wu et al., 2019).These bacteria may turn out to be new candidate phylotypes for predicting and treating renal dysfunction in DN.

Our study has a number of limitations. Our study did not measure resveratrol or its metabolites in the fecal slurries used for FMT studies, thus it remains possible that the beneficial effects of the FMT observed in our study was confounded by residual resveratrol. In addition, we used fecal samples from db/m mice as a donor for our FMT studies. Future studies will need to be conducted to compare beneficial effects of FMT from RSV-treated db/db and db/dm mice. Nevertheless, the results from our studies suggest that So changing the fecal microbiota composition by resveratrol is only one of a variety ofis one mechanism by which resveratrols that protects against the development of DN (Wenzel and Somoza, 2005; Kim et al., 2018). However, in human study, the specific role of resveratrol on the gut microbiota and the development of DN are not yet clear. More clinic trials are needed to confirm these and other possible effects and mechanisms.

## Conclusions

Resveratrol treatment is a potentially beneficial therapeutic intervention against the progression of DN, by improving the gut environment and reducing the inflammation response, and that these changes may be an important mechanism by which resveratrol mediates its beneficial renal function effects. These findings provide supporting evidence for the gut–kidney axis in DN.

## Data Availability Statement

The datasets generated for this study can be found in NCBI BioProject PRJNA628114.

## Ethics Statement

The animal study was reviewed and approved by Nanjing Medical University and approved by the Institutional Animal Care and Use Committee of Nanjing Medical University, No. 14030134.

## Author Contributions

Conceptualization, D-FD, XL, and Y-BL. Methodology, T-TC, X-LY, R-RL, HC, Y-YW, WL. Software, X-YL, M-LP. Validation, YT and X-LY. Formal Analysis, D-FD, T-TC, X-LY. Investigation, H-JY. Resources, HM and Y-BL. Data Curation, Y-BL, T-TC, and X-LY. Writing—Original Draft Preparation, D-FD, T-TC. Writing—Review and Editing, X-YL, AS, J-HM. Supervision, T-TC. Project Administration, D-FD. Funding Acquisition, D-FD.

## Funding

This work was supported by the grants from Major Research and Development Project of Jiangsu (BE2016800), Jiangsu Youth Medical Talents Project (QNRC2016674) and Nanjing Science Technology Plan Project (201715015).

## Conflict of Interest

The authors declare that the research was conducted in the absence of any commercial or financial relationships that could be construed as a potential conflict of interest.

## Supplementary Material

Table S1 Alpha-diversity index in db/m groups, db/m+Res, db/db, db/db+Res mice.

Table S2 Relative gut microbiota abundance at the genus level in four groups.

Table S3 Alpha-diversity index in db/db mice following control-FMT (n = 8) or resveratrol-FMT (n = 8).

Table S4 Relative species abundance at the genus level in control-FMT (n = 8) or resveratrol-FMT (n = 8) mice

Figure S1 Bacterial composition at the species level of each sample in four groups. Gut microbe relative abundance at the species level of each sample in db/m, db/m+Res, db/db, and db/db+Res mice.

Figure S2 Microbiome composition in control-FMT and resveratrol-FMT db/db mice. The heatmap of relative species abundance at the genus level in control-FMT (n = 8) or resveratrol-FMT (n = 8) db/db mice.
