# Supplementary figures and images for: Resveratrol Modulates the Gut Microbiota and Inflammation to Protect Against Diabetic Nephropathy in Mice
Source: Front Pharmacol. 2020 Aug 19;11:1249. doi: 10.3389/fphar.2020.01249 (PMC7466761; doi:10.3389/fphar.2020.01249)

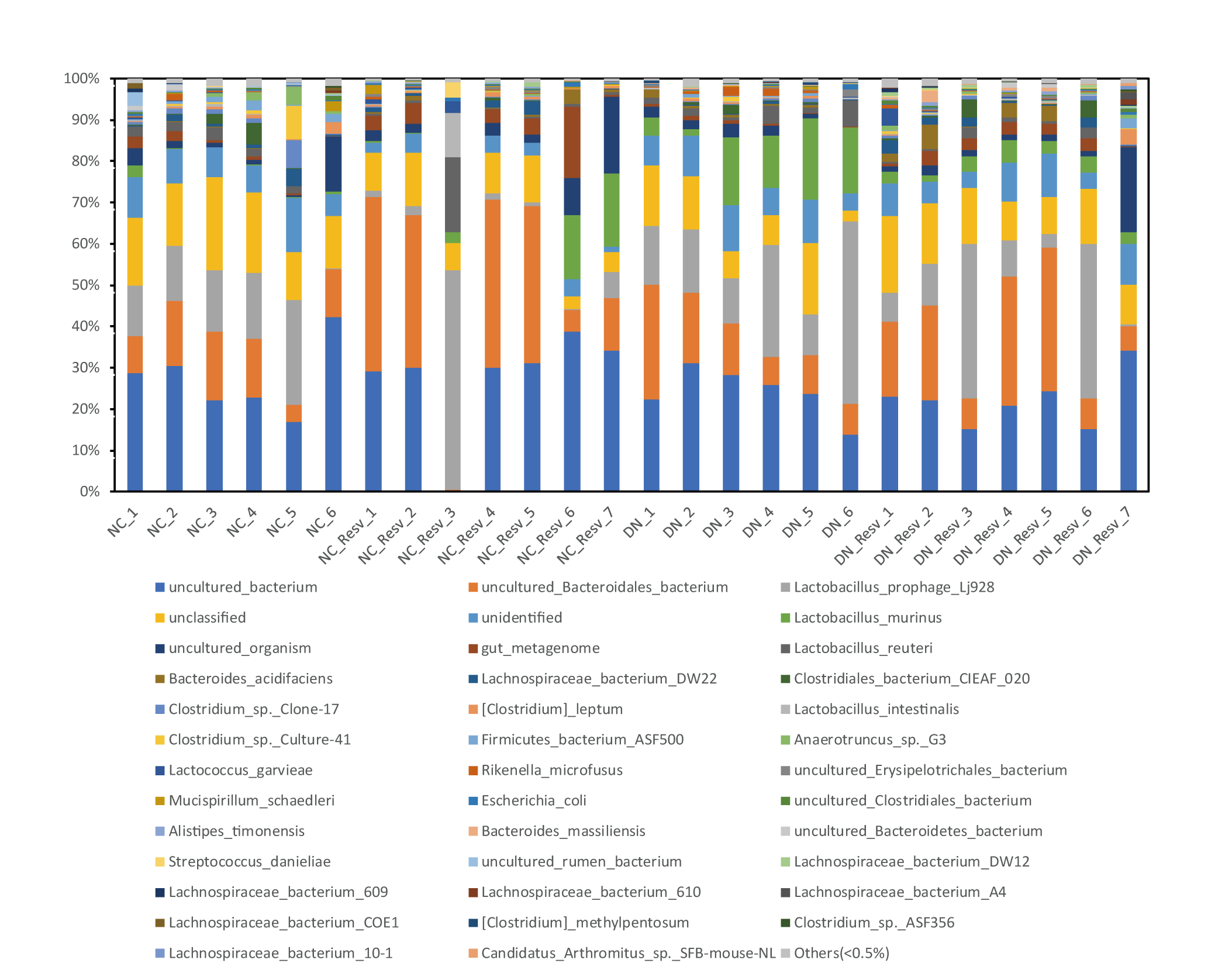

Supplement: Figure S1 — Bacterial composition at the species level of each sample in four groups. Gut microbe relative abundance at the species level of each sample in db/m, db/m+Res, db/db, and db/db+Res mice. [file Image_1.tif]

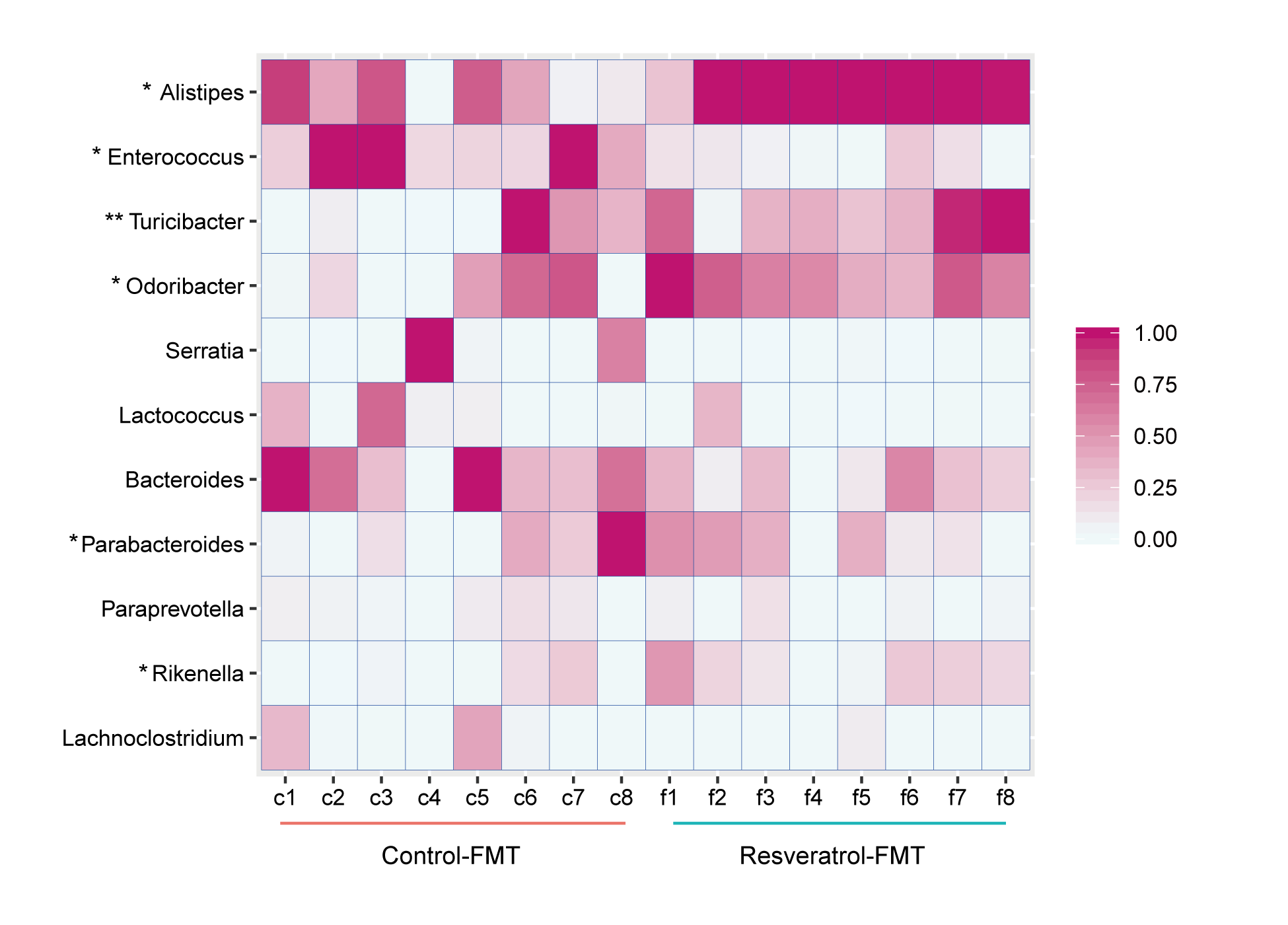

Supplement: Figure S2 — Microbiome composition in control-FMT and resveratrol-FMT db/db mice. The heatmap of relative species abundance at the genus level in control-FMT (n = 8) or resveratrol-FMT (n = 8) db/db mice. [file Image_2.tif]
